# Alcohol Mixed with Energy Drinks (AmED) Use among University Students: A Systematic Review and Meta-Analysis

**DOI:** 10.3390/nu14234985

**Published:** 2022-11-24

**Authors:** Andrea De Giorgi, Federica Valeriani, Francesca Gallè, Francesca Ubaldi, Annalisa Bargellini, Christian Napoli, Giorgio Liguori, Vincenzo Romano Spica, Matteo Vitali, Carmela Protano

**Affiliations:** 1 Department of Public Health and Infectious Diseases, Sapienza University of Rome, 00185 Rome, Italy; 2 Department of Movement, Human, and Health Sciences, University of Rome “Foro Italico”, 00135 Rome, Italy; 3 Department of Movement Sciences and Wellbeing, University of Naples “Parthenope”, 80133 Naples, Italy; 4 Department of Biomedical, Metabolic and Neural Sciences, Section of Public Health, University of Modena and Reggio Emilia, 41125 Modena, Italy; 5 Department of Medical Surgical Sciences and Translational Medicine, “Sapienza” University of Rome, 00189 Rome, Italy

**Keywords:** energy drink, alcohol, undergraduates, adverse effects, health-related behaviors

## Abstract

In the last decades, there has been a huge increase in the consumption of both Energy Drinks (EDs) and alcohol and, concurrently, these two trends generated the additional practice of mixing ED with alcohol, known as Alcohol mixed with Energy Drink (AmED). One of the most important group of AmED consumers is represented by young. Indeed, the study population of the researches in this field are mainly represented by college students and the results evidence a great range of negative consequences for health. The purpose of the systematic review was to explore the prevalence of AmED consumption among undergraduate students, together with motivations to their use, adverse effects and health-related behaviors associated to AmEDs use. The review was conducted according to the PRISMA Statement and PubMed, Scopus and Web of Science were interrogated. 42 articles, published from 2008 to 2021, were included in the review. An overall prevalence rate of 37% was estimated for AmEDs use in undergraduates, with geographical differences. Although a decrease in consumption was observed throughout the studied period, a continuous monitoring on this phenomenon is needed for identifying those risk groups that could develop risky behaviors related to AmEDs consumption and provide them targeted educational interventions.

## 1. Introduction

The consumption of Energy Drinks (EDs) is very popular because of their advertised effects such as the increase of energy, concentration, athletic performance, metabolism and stimulation of mental activity and alertness, which represent the main reasons to use these beverages, other than liking the taste [1,2,3,4]. One of the most relevant group of consumers is represented by students, which use EDs to overcome the high levels of stress related to study commitments [5] and to get benefits in cognitive performance, concentration and mood [6] as well as to stay awake and increase energy levels. After more than 20 years since the debut of the first ED company, there has been a huge increase in their consumption, complemented by an overall increase in alcohol intake [7], and concurrently these two trends generated the additional practice of mixing ED with alcohol, which is known as the consumption of Alcohol mixed with Energy Drink (AmED). Compared with alcohol alone, consuming AmED increases total alcohol consumption; one hypothesis which justifies this increase is that the stimulant effects of caffeine, one of the main ingredients of EDs, may counteract the depressant effects of alcohol [4]. Indeed, most of the research comparing AmED consumers with alcohol only consumers reported higher levels of alcohol in AmED consumers respect to alcohol users. Usually, the target of these studies is the university students’ population [8], emphasizing how dangerous are alcohol-related negative consequences and how susceptible is the undergraduate population. Alcohol consumption among college students contributes to a range of negative alcohol-related consequences; for example, in the United States each year, approximately 1825 deaths, 97,000 sexual assaults, and 600,000 injuries are alcohol-related [9]. In South Korea, around 10.8% of deaths among college students are attributed to alcohol, while more than 50% of colleges experience alcohol-related problems like campus vandalism and violence by intoxicated students [10]. The risks are even greater with the consumption of AmEDs, which has been associated with increased odds of driving a car under their influence, being hurt or injured, experiencing unwanted sexual contact, having unprotected sex, and using drugs [11]. Then again, heavy consumption of AmEDs may lead to alcohol addiction, liver diseases, and obesity or diabetes related with the high-caloric content of these drinks. The caffeine content of EDs increases the need for more alcohol consumption, leading frequently to alcohol intoxication [12], not to mention all other caffeine adverse effects, such as to anxiety, irritability, restlessness, sleep disorders, gastrointestinal upsets, tremors, tachycardia, and psychomotor agitation [13,14]. According to so many side effects, risky behaviors and health outcomes, there is a need for an appropriate and detailed assessment of this issue throughout scientific literature, to estimate consumption prevalence and to assess the reasons for their use, the associated health-related behaviors and their adverse effects, especially in high-risk groups such as university students. Moreover, several surveys have been performed in different countries to evaluate alcohol-related impact, instead of a fair amount for energy drinks related issues, but there are very few studies concerning AmED consumption which go beyond the simple comparison between AmED and alcohol only.

The purpose of the present systematic review was to explore the prevalence of AmED consumption among undergraduate students, analyzing the data coming from the available literature on this topic. A meta-analysis was also performed on these data to estimate the overall prevalence of undergraduates’ AmEDs use worldwide. Furthermore, adverse effects and health-related behaviors associated to AmEDs consumption and motivations to their use in this population group were also systematically analyzed.

## 2. Materials and Methods

### 2.1. Selection Protocol and Search Strategy

This systematic review was performed according to the Preferred Reporting Items for Systematic Review and Meta-Analysis (PRISMA) guidelines [15]. The review protocol was registered in PROSPERO (reference number CRD42022328431).

The review question was framed using PICOS framework and the eligibility criteria selected for the present systematic review are reported in Table 1. A university student was considered as a student enrolled in a college or university. The range of age of university students was 18–24 years. However, in some of the studies no age limits were used and then also older students were added in the review.

Three electronic databases (PubMed, Scopus and Web of Science) were questioned using the following query string: “energy drink” AND “alcohol” OR “AmED” AND (“university students” OR “college students” OR “undergraduate*”). Table S1 shows the detailed search strategy.

The search was performed from 20 to 31 May 2022 and was carried out by title, abstract, and MeSH terms on PubMed or keywords on Scopus and Web of Science.

Table 2 shows the inclusion and exclusion criteria used in the selection process.

Titles and abstracts obtained from the databases were transmitted to the reference software Zotero systematic review manager for the process of assessment. The subsequent step was screening by title and abstract the potentially eligible studies, following the inclusion criteria; the screening was performed by five authors (F.G., C.P., F.V., A.D.G.) independently. Then, full texts were read independently by the consensus team (F.G., C.P., F.V., A.D.G.) and disagreements about their inclusion were achieved by consensus among the authors.

### 2.2. Data Extraction Process and Quality Assessment

A specific set of categories were chosen as the extracted data following consensus of all authors: bibliographic information like author, year, country, sample size, study subject/population with age and gender; and AmED consumption estimate, associated health-related behaviors, reason and adverse effects due to AmED consumption as outcomes.

The Newcastle-Ottawa Quality Assessment Scale—NOS—adapted for cross-sectional studies was used for quality assessment. An overall quality rating was assigned to each eligible article according to the number of criteria met as follows: Good Quality (all criteria met, low risk of bias); Fair Quality (1 criteria not met or 2 criteria unclear, moderate risk of bias); Poor Quality (2 or more criteria not met, high risk of bias). Five authors (F.G., C.P., F.V., A.D.G.) independently assigned a score to each study, and disagreements were settled by consensus among all the authors.

### 2.3. Statistical Analysis

Comprehensive Meta Analysis 4.0 (Biostat, Englewood, NJ, USA) was used for meta-analysis and statistical elaborations. The prevalence of AmED consumption and 95% CI were extracted for each study. The pooled estimation of prevalence was calculated using random-effects model for higher external validity of findings because are included studies with different populations. According to previous studies [16,17], the formula Logit = Ln(p/(1 − p)), where p represents the prevalence rate and Ln the natural logarithm, was used to transform prevalence rate in its logarithmic form, and V(Logit) = 1/np + 1/n(1 − p), where V represents the variance, was used to transform samples’ variance. The conversion is based on the formula p = eLogit/(eLogit + 1), with e being the base of the natural logarithm.

In order to evaluate the heterogeneity of the selected studies, the I^2^ test and the classical measure of heterogeneity Cochran’s Q (Hedges Q statistic) were used. The following thresholds of I^2^ were employed: <25% = low heterogeneity; <50% = moderate heterogeneity; and >75% = high heterogeneity [18]. To assess the publication bias, the Egger’s test and Funnel plot were employed [19]. Meta-regression and subgroup analyses were performed to evaluate the sources of heterogeneity [20,21,22]. For meta-regression analysis, sample size, gender and age of participants, WHO Regions location (European Region, African Region, Region of the Americas, South-East Asia Region, Eastern Mediterranean Region, Western Pacific Region), years since publication and methodological quality of the studies were considered as possible sources of heterogeneity; the time considered to assess AmED consumption was also included into the meta-regression analysis by considering four subgroups (Past week, Past 30-days, Past 60-days, Past 90-days and Past year). Three articles [23,24,25] were excluded from the meta-analysis and meta-regression analyzes because they did not report the prevalence rate of AmED consumption. Two other papers were excluded because they involved the same cohort of participants [26].

## 3. Results

### 3.1. Article Selection

**Figure 1:**
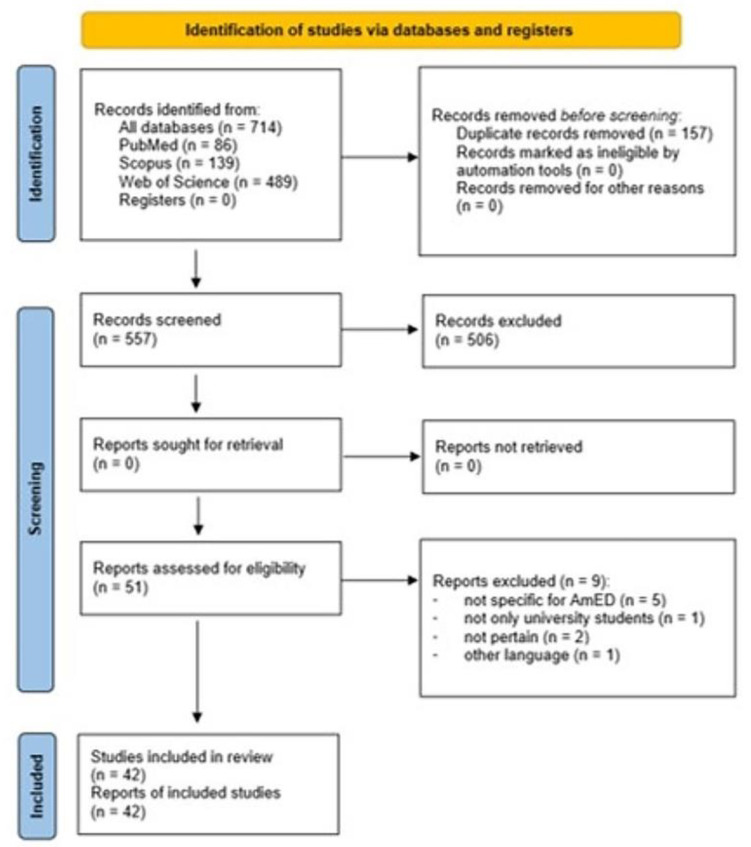
PRISMA flowchart of search strategy.

Figure 1 shows the steps of the article selection process used for the systematic review following the PRISMA statement [15].

On a total of 714 records found, 557 were screened for inclusion and 51 assessed for eligibility. Five articles were excluded because not specific for AmED, one article because the study population did not include only university students, two articles because they were not pertinent, and one article because it was not in English. Finally, 42 articles met the inclusion criteria and were included in the analysis (Table 3 and Table 4).

### 3.2. Characteristics of the Selected Studies

The included articles were published between 2008 and 2021 and performed in several countries. All the studies included male and female subjects with a range of 18–34 years; the sample size ranged from 72 [56] to 10,340 individuals [60]. The evaluation of ED consumption frequency is based mainly on a monthly report [26,30,33,35,38,39,41,45,49,50,52,54,55,59,61,63], less commonly on a yearly report [29,34,37,42,53,60,62,64] and on a weekly report in only two studies [23,54]. Thirteen articles analyzed AmED consumption in comparison with the consumption of alcohol alone [24,33,34,35,36,37,38,39,41,43,45,46,53] while four articles compared the AmED consumption with EDs only [28,29,42,44].

As for the quality assessment, a total of 25 articles showed a poor quality rating, which means with important limitations that could invalidate the results and high risk of bias in general [23,24,25,26,27,31,34,36,38,40,41,43,44,45,46,47,48,55,56,57,58,60,61,62,63,64]. A total of 15 assessed studies reported a fair quality rating, with no known important limitation that could invalidate the results and with a moderate or acceptable risk of bias [28,29,30,35,37,42,49,50,51,52,53,54,58,61] and only two studies reported a good quality rating with all criteria met and a very low risk of bias [32,33].

### 3.3. AmED Consumption Prevalence and Related Aspects

Among the selected studies, the prevalence of AmED users ranges from 25.6% [37] to 84.4% [32]. Five articles considered the differences of consumption according to gender [36,41,53,57,63], showing no significant differences with the exception of the study by Woolsey et al. [64], which found a major intake in males.

The adverse effects related to AmED consumption were investigated in 20 out of the 42 selected articles. Endanger themselves by driving [28,31,37,38,39,41], high propensity for sensation seeking, impulsiveness, interpersonal consequences and having arguments or fights [29,30,37,39,41,42] and headache or dizziness [43,46,49,51] were mainly reported by participants. Other quite frequently reported adverse effects were difficulty to limit the alcohol quantity or binge drinking [30,32,38,39,41], unwanted or particular sex behaviors [30,31,39,43], depression symptoms, anxiety, suicidal thoughts or attempts [32,34,53,62] and sick stomach [26,43,46,49]. Other less reported adverse effects were been hurt or injured [31,33], disturbance in sleep [34,62], decrease in quality of work or study [30,39], put on weight [30,34] and memory loss [49].

Among the studies assessed, 23 analyzed the prevalence of health-related behaviors associated with AmED consumption. The most frequently reported behaviors were hazardous drinking and heavier alcohol intake [25,27,28,32,55,56,58,61,62], drunk driving while knowingly over the blood alcohol content driving limit and risk taking with frequent injuries [43,53,54,63,64] and sexual risk-taking behaviors [46,49,50,52]. Other less frequently reported behaviors were increased smoking habit [30,60], increased drug consumption [33,56] and even a positive associated behavior reported such as happiness and fun [24,51].

Only thirteen studies investigated reasons behind the AmED consumption, revealing so many of them with a common thread: to get drunk [30,35,40,42,48,63], to reduce negative effects of alcohol, to relax or to enjoy at parties [23,30,42,57,64], to hide the alcohol’s flavor or reduce sedation of alcohol alone [35,48], to treat hangover [34,62,63]; other reasons instead were more wider, such as to celebrate [23,30,40], for common availability [38], to keep themselves awake and increase alertness [34,35,38,45,62] and finally because they simply liked the taste [30,40].

### 3.4. Meta-Analysis and Meta-Regression Results

**Figure 2:**
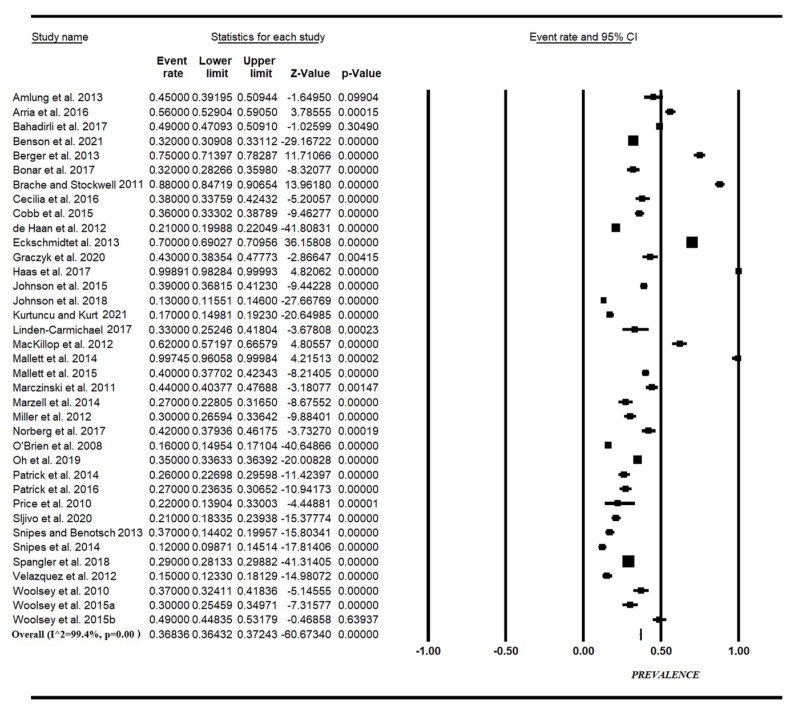
Forest plot for the prevalence of AmED consumption in students [27,28,29,30,31,32,33,34,35,36,37,38,39,40,41,42,43,45,46,47,48,49,50,51,52,53,54,55,56,57,58,59,60,61,62,63,64,65].

With regards to the meta-analysis results, the estimated overall prevalence of AmED consumption (Figure 2) was 37% in undergraduate students (95% CI, the range of prevalence is 13–99%), with significant heterogeneity among studies (Q test: *p* < 0.001; I^2^ = 99.4%). Sensitive analysis did not substantially change the pooled prevalence of AmED consumption, which resulted equal to 38% (95% CI: 13–99%) with the inclusion of Linden-Carmichael et al., 2017, 2018 [43,44], suggesting that no one single study had a disproportional impact on overall prevalence.

A visual inspection of funnel plot suggested no publication biases in the present study (*p* = 0.001) (Figure S1).

To investigate the role of the study characteristics in influencing the heterogeneity of the global prevalence of AmED consumption, a meta-regression analysis was performed considering sample size, gender, age, publication year, time to which AmED consumption was referred to, methodological quality of the study and WHO region in which the study was performed. The results showed that the prevalence of AmED consumption was independent by the amount of females in the sample (*p* = 0.750), mean age (*p* = 0.140), time of AmED consumption (*p* = 0.240), or methodological quality (*p* = 0.250). Instead, the prevalence of AmED consumption in the world slightly showed a decrease with the increase of the year of publication (*p* < 0.05). According to the meta-regression analysis, the study location showed a slight action of moderation (Q = 3.19, df = 2, *p* < 0.05). The prevalence of AmED consumption was 73% in AMR Region, 19% in EUR, 8% in WPR.

## 4. Discussion

Mixing Alcohol with Energy Drinks is a common practice among young people and a great concern for public health because it can be associated with several adverse effects and other risky habits, such as binge drinking and alcohol dependence [4,7,8,28,65]. To explore the prevalence of AmED consumption and motivations to their use among undergraduate students, the available literature from 2008 to 2021 was analyzed in this review. The results showed that the estimated overall prevalence of AmED consumption was 37% in the populations studied, with a great variability and a significant heterogeneity among the studies.

Among the possible factors influencing the heterogeneity of the global prevalence of AmED consumption arose the study location, with AMR and EUR regions showing the major prevalence. However, it should be noted that the data reported by the National Institute on Drug Abuse at The National Institutes of Health in USA showed a substantial decline in lifetime alcohol use among youths from 1991 to 2017 [64]. These data are in agreement with the observation that the year of publication is negatively correlated with the prevalence of AmED consumption, and recent studies reported a lower prevalence of AmED among the undergraduate students compared to older ones. Probably, the campaigns implemented to contrast addictions had influence also on the consumption of AmED [64]. However, the overall prevalence of AmED consumption worldwide is still not negligible, especially considering that the studied consumers are young. Besides, the AmED consumption can determine the increase of other risk factors, as hazardous drinking, defined by WHO as a pattern of alcohol consumption that increases the risk of harmful consequences for the user or others [65], and heavier alcohol intake and consequently drunk driving [25,26,27,28,32,53,54,55,56,58,61,62,63,64], sexual risk-taking behavior, smoking and drug consumption [24,30,33,46,49,50,51,52,58], which can be threatening for health and life.

In addition, participants have reported several adverse effects such as high propensity for sensation seeking, impulsiveness, interpersonal consequences and having arguments or fights and headache or dizziness. Other frequently reported adverse effects were difficulty to limit the alcohol quantity or binge drinking. Particularly, binge drinking is defined as consuming 5 or more drinks on an occasion for men or 4 or more drinks on an occasion for women [66]. It is a harmful risk behavior related with serious injuries and several diseases and with an increased risk of alcohol use disorder [67].

Moreover, it should be considered that the quarantines occurred during the COVID-19 pandemic had significantly changed alcohol use in many countries, shifting places of consumption from bars and restaurants to home, mainly thanks to a widely increasing e-commerce. As reported by the Organisation for Economic Co-operation and Development, alcohol sales increased by 3% to 5% in Germany, the United Kingdom and the United States in 2020 compared to 2019 [68]. Even though many individuals reported a decrease or no change in their consumption behaviors during the lockdowns, there has been an increase in frequency and quantity of alcohol use, especially among women, parents of young children, people with higher income and those with anxiety and depressive symptoms in many countries. Some of the problems associated with harmful alcohol consumption and risky behavior such as binge drinking were intensified by the pandemic, even though the long-term impacts of COVID-19 on alcohol consumption are not completely known so far [68,69]. Therefore, it is possible that the pandemic and related control measures have exacerbated even the use of AmEDs, especially among specific population groups, leading to different prevalence rates.

For these reasons, it is essential to understand the consumption motivations and also the possible consequences of AmEDs use, in order to structure new health programs to counteract this phenomenon. AmEDs consumption seems to be related with neuroendocrine-independent brain stress systems, that influence drinking behavior in a dynamic and complex manner [70,71]. Thus, just like the excessive alcohol use as a common response to stress is caused by several triggers, including psychological motivations, as boredom, disruption to routines, distress, also the AmED consumption can be triggered by these reasons [72,73]. However, also physiological causes, such as nutritional deficiencies, dehydration, hormonal changes or the activation of reward-related brain areas, can determine their consumption [74]. Among the examined studies only thirteen investigated the reasons behind the AmED consumption, revealing several common threads beyond to get drunk: to reduce negative effects of alcohol, to relax or to enjoy at parties [23,30,42,57,63] to hide the alcohol’s flavor or reduce sedation of alcohol alone [35,48] to treat hangover [34,62,63]. A non-negligible percentage of university students consume AmEDs to hide the flavor of alcohol, to drink more and feel less drunk and this data is in line with the literature [48,52]. Indeed, energy drinks may alter the effects of alcohol through the inhibition of dopamine transmission [75,76]. This aspect is a concern and underlines the tight association between AmEDs and alcohol use.

There are several limitations to our analysis. First of all, the data in each selected study were obtained by self-report survey and recall bias may have interfered with obtaining reliable information. In addition, the heterogeneity of recall period and measures of consumption frequency may represent other biases. Above all, the quality of the majority of the studies was not good, which affect the validity of our findings. However, this review offers a systematic picture of AmEDs consumption and related aspects worldwide, together with the strength of a meta-analytic analysis. In order to reduce the effects of articles’ heterogeneity, the meta-regression analysis was controlled for sample size, geographical location, year of publication, time chosen for reporting AmEDs consumption and methodological quality of the studies, together with gender and age of participants. Notwithstanding the reported variability, it seems that only the time of publication and the study location had a slight effect on the results.

## 5. Conclusions

Our findings show that a global prevalence rate of 37% is estimated for AmEDs use in undergraduates, with geographical differences. Although a decrease in consumption was observed throughout the period in which the selected studies were published, a continuous monitoring on this phenomenon is needed in order to identify those risk groups that could develop risky behaviors related to AmEDs consumption and provide them targeted educational interventions. The creation of ad hoc surveillance systems could help healthcare systems in controlling the risks possibly related with AmEDs use.

## Figures and Tables

**Table 1:** Eligibility criteria used in the article selection process based on the PICOS framework.

	Eligibility Criteria
Population	University students, independently by their gender and age
Intervention	Consumption of AmEDs in everyday life
Comparison	Age-, gender- and condition-matched control group (if present)
Outcomes	To explore the prevalence of AmED consumption; adverse effects and health-related behaviors associated to AmEDs consumption and motivations to their use were also analyzed
Study design	Cross-sectional studies

**Table 2:** Inclusion and exclusion criteria used in the article selection process.

Inclusion Criteria	Exclusion Criteria
Articles reporting data about AmED specific consumption of university students, independently by their gender and age	Articles presenting studies that included individuals who were not university students or studies which regarded the consumption of other dietary supplements than AmEDs or only EDs or alcohol alone or other substances
Articles presenting cross-sectional studies	Articles presenting clinical trials, experimental studies, reviews, meta-analysis, case studies, proceedings, qualitative studies, editorials, commentary studies and any other type
Articles published in English language, from the inception to 31 May 2022	Articles published in languages other than English

**Table 3:** Characteristics of the samples and main results related to Alcohol mixed with Energy Drinks (AmEDs) patterns of consumption in the selected studies.

Author Year Country	Sample Size Age (Mean Value ± SD and/or Range) Gender (%)	AmED Consumption Estimate	Adverse Effects	Quality Assessment (NOS)
Amlung et al., 2013 USA [27]	273; 20.0 ± 1.7 years; 73% Female, 27% Male	45% in the last month occasional use. 1–2 days: 25%; 3–5 days: 11%; 6–9 days: 5%; 10–14 days: 3%; 15–19 days: 1%	NR	Poor (4)
Arria et al., 2016 USA [28]	1000; 22–25 years; 45% Male, 55% Female	71% drank AmED and 85% drank ED alone; 56% both styles	More frequent drunk driving	Fair (5)
Bahadirli et al., 2017 Turkey [29]	2632; 23.43 ± 2.27 years; 51.2% Male, 48.8% Female	In the last year 65.2% consumed alcohol only, 59.9% ED only, 48.9% AmED. 52.2% consumed AmED in the last 30 days	Higher propensity for sensation seeking and impulsiveness	Fair (5)
Benson et al., 2021 Australia [30]	6881; 21.3 ± 2.6 years; 41.0% Male, 59% Female	Mean (SD) of 1.7 (2.2) AmED consumption days per month The number of energy drinks mixed with alcohol: one 250 mL can (57.8%), two cans (22.7%), three cans (9.2%), more than three 10.3%	Impulsiveness, decrease in quality of work or school work, risky sexual behavior, difficult to limit amount of alcohol, overweight	Fair (5)
Berger et al., 2013 USA [31]	606; 21.5 ± 1.7 years; 38.4% Male, 61.6% Female	64.7% consumed AmED in the last year	Driven a car while under the influence (36.4%); been hurt or injured (16%); and experienced unwanted sexual contact as a result of consuming alcohol (9.1%)	Poor (4)
Bonar et al., 2017 USA [32]	560; 19.57 ± 1.75 years; 33% Male, 67% Female	84.4% consumed AmED rarely, 10.0% AmED use half the times they drink alcohol, 5.6% AmED every time they drink alcohol.	Increase in smoking, alcohol and drug use, symptoms of depression	Good (7)
Brache and Stockwell 2011 Canada [33]	465; 24.03 ± 6.7 years; 44% Male, 56% Female	88% consumed alcohol and 26% consumed AmED 39% in the last 30 days	They were more likely to have ridden home with a driver who had been drinking, driven home after drinking and been hurt or injured	Good (7)
Cecilia et al., 2016 Italy [34]	479; 22.3 ± 4.4 years; 36% Male, 64% Female	65% consumed AmED and 27% are classified as regular AmED users	Overweight, sleep disorders, trait anxiety	Poor (4)
Cobb et al., 2015 USA [35]	1174; 75.3% 18–19 years old; 35.5% Male, 64.5% Female	34% consumed AmED and 36% alcohol only in the last 30 days. Average days drinking alcohol-caffeine per month 3.6 (SD 3.5)	NR	Fair (5)
de Haan et al., 2012 Netherlands [36]	6002; 22.1 ± 2.6 years; 32.5% Male, 67.5% Female	26% consumed AmED	NR	Poor (4)
Eckschmidtet al., 2013 Brazil [37]	8672; 58.9% 25–34 years old; 40% Male, 60% Female	74.4% consumed alcohol only and 25.6% AmED in the last year. Frequency of alcohol use in the last year: daily 56.5%, weekly 40.5%, monthly 90.1%	Driving unbelted (28.2%), driving at high speed (38.1%), having been fined for any reason (10.8%), having had arguments or fights while in traffic (9%), drinking and driving (24.6%), driving after binge drinking (22.1%), riding with an intoxicated driver (32.9%)	Fair (5)
Graczyk et al., 2020 USA [38]	422; 18–26 years old; 41.23% Male, 58.77% Female	21.8% consumed AmED in the last 2 months	Driving while intoxicated, higher occasions of binge drinking, higher alcoholic drinks per occasion	Poor (4)
Haas et al., 2017 USA [39]	458; 19.30 ± 1.86 years; 39.9% Male, 60.1% Female	65% consumed AmED in the last 2 weeks	Higher interpersonal consequences, engagement in risky behaviors, academic difficulties, impaired control while drinking, more physiological dependency symptoms	Fair (5)
Johnson et al., 2015 UK [40]	1873; 20.9 ± 2.20 years; 44.6% Male, 55.4% Female	39.1% consumed AmED	NR	Poor (4)
Johnson et al., 2018 UK [41]	1873; 20.5 ± 2.00 years; 50.8% Male, 49.2% Female	15.2% consumed AmED	Driving while intoxicated, higher occasions of binge drinking and impulsiveness	Poor (4)
Kensinger et al., 2014 USA [23]	540; 20.14 ± 1.59 years; 35% Male, 65% Female	NR	NR	Poor (4)
Kurtuncu and Kurt 2021 Turkey [42]	1202; 20.82 ± 1.86 years; 37.8% Male, 62.2% Female	17.3% consumed AmED in the last year and 11.6% in the last 30 days	Higher alcohol intake and impulsiveness	Fair (5)
Lau-Barraco et al., 2013 USA [24]	583; 19.84 ± 1.69 years; 31% Male, 69% Female	6% consumed 16 AmED, 5.2% 8 AmED, 23% 4 AmED and 65.9% less than one AmED weekly	NR	Poor (4)
Linden-Carmichael and Lau-Barraco 2017 USA [26]	122; 20.39 ± 2.08 years; 26.2% Male, 73.8% Female	26% consumed AmED	Sexual risk behaviors, hangover, headache, sick stomach, vomiting, less energy	Poor (4)
Linden-Carmichael and Lau-Barraco 2017 USA [43]	122; 20.39 ± 2.08 years; 73.8% Female 26,2% Male	26% consumed AmED	Headache, sick stomach	Poor (3)
Linden-Carmichael and Lau-Barraco 2018 USA [44]	122; 20.39 ± 2.08 years; 73.8% Female 26.2% Male	26% consumed AmED	NR	Poor (3)
MacKillop et al., 2012 USA [45]	409; 20.13 ± 1.88 years; 71% Female 29% Male	62% consumed AmED, 48% in the last 30 days	NR	Poor (3)
Mallett et al., 2014 USA [46]	195; 21 ± 0.27 years; 43% Female 57% Male	11.8% (Moderate drinker, low proportion AmEDs); 70.5% (Moderate drinker, high proportion AmEDs); 8.0% (Heavy drinker, low proportion AmEDs); 97.8% (Heavy drinker, high proportion AmEDs)	Headache, hangover, vomiting	Poor (4)
Mallett et al., 2015 USA [47]	1710; 18.56 ± 0.50 years; 57.7% Female 42.3% Male	39.6% consumed AmED	NR	Poor (4)
Marczinski et al., 2011 USA [48]	706; 20.9 ± 5.3 years; 49.9% Female 50.1% Male	44.0% consumed AmED, 9.3% in the last 2 weeks	NR	Poor (2)
Marzell et al., 2014 USA [49]	386; 18 ± 0.45 years; 59% Female 41% Male	27% consumed AmED in the last 30 days	Headache, sick stomach, memory loss	Fair (6)
Miller et al., 2012 USA [50]	648; 20.14 years (range 18–40); 47.5% Female 52.5% Male	29.3% consumed AmED in the last 30 days	NR	Fair (6)
Norberg et al., 2017 Australia [51]	549; 19.21 ± 1.46 years; 70% Female 30% Male	42% consumed AmED in the last 90 days	Dizziness, ill	Fair (5)
O’Brien et al., 2008 USA [52]	4271; 20.4 ± 2.8 years; 61% Female 39% Male	16.3% consumed AmED in the last 30 days	Dizziness, fatigue, headache, trouble walking	Fair (6)
Oh et al., 2019 South Korea [53]	4592; 50.8% Female 49.2% Male; age range not reported.	22.0% of alcohol-consuming men and 13.4% of alcohol-consuming women reported AmED consumption in the last year	Depressive thoughts, suicidal thoughts, suicidal attempt	Fair (5)
Patrick et al., 2014 USA [54]	620; 19.49 ± 0.43 years; 51% Female 49% Male	26% consumed AmED in the last 30 days and 29% reported frequent use	NR	Fair (6)
Patrick et al., 2016 USA [55]	614; 19.5 ± 0.43 years, 18.0–21.75 years; 47% Male, 53% Female	27% consumed AmED in the last 30 days	NR	Poor (3)
Price et al., 2010 Canada [56]	72 ED users; 17–29 years; 43% Male, 57% Female	76% consumed AmED, 53% in the last week	NR	Poor (2)
Sheehan et al., 2016 USA [25]	733 alcohol consumers; 20.21 ± 3.56 years, 18–47 years; 32.4% Male, 67.6% Female	7.87 AmEDs per week	NR	Poor (3)
Sljivo et al., 2020 Bosnia and Herzegovina [57]	812; 21.37 ± 1.98, 18–38 years; 26.9% Male, 73.1% Female	21.5% consumed AmED in the last year: 67.3% rarely, 15.9% once or twice a month, 11.2% once a week, 4.7% 2 to 3 days a week; 72.9% 1–2 AmED in a single session, 14.0% 3–4, 2.8% 5–6, 2.8% 7–8, 4.7% 9–10	NR	Poor (4)
Snipes and Benotsch, 2013 USA [58]	704; 19.0 ± 11.80 years; 40.1% Male, 59.9% Female	17.2% consumed AmED in the last 30 days	NR	Fair (5)
Snipes et al., 2014 USA [59]	757; 18.90 ± 1.51 years, 18–25 years; 31.2% Male, 68.8% Female	11.6% consumed AmED in the last 30 days, 9.7% in the last week	NR	Poor (4)
Spangler et al., 2018 USA [60]	10,340; 92.4% 18 years, 7.6% > 18 years; 37.04% Male, 62.96% Female	29.4% consumed AmED in the last year	NR	Poor (3)
Velazquez et al., 2012 USA [61]	585; 18.7 years; 64% Male, 56% Female	14.9% consumed AmED in the last 30 days	NR	Fair (5)
Woolsey et al., 2010 USA [62]	401; 19.8 years; 64.1 Male, 35.9% Female	37.4% consumed AmED in the last year	Sleep disturbance, nervousness and rapid heartbeat were more common in AmED than in alcohol-only consumers	Poor (3)
Woolsey et al., 2015 USA [63]	355 alcohol users	30.1% consumed AmED in the last 30 days	NR	Poor (3)
Woolsey et al., 2015 USA [64]	549 alcohol users; 22.01 ± 4.160; 32.1% Male, 67.9% Female	48.8% consumed AmED in the last year, mainly men (*p* < 0.001)	NR	Poor (4)

NR = Not Reported.

**Table 4:** Health-related behaviors and motivation associated with Alcohol mixed with Energy Drinks (AmEDs) consumption in the selected studies.

Author Year Country	Associated Health-Related Behaviors	Reason
Amlung et al., 2013 USA [27]	Greater levels of hazardous drinking above and beyond the influence of collateral risk factors such as impulsivity and demand for alcohol	NR
Arria et al., 2016 USA [28]	Heavier alcohol use, higher risk for drunk driving relative to individuals who consumed ED exclusively with alcohol (41.3% vs. 28.7%)	NR
Bahadirli et al., 2017 Turkey [29]	NR	NR
Benson et al., 2021 Australia [30]	AMED consumers were significantly more often smokers and had higher risk-taking scores	I like the taste, I wanted to drink something else, to get drunk, to celebrate a special occasion, I received the drink from someone else (and did not want to refuse it), it feels like it reduces the negative effects of alcohol, because others drink it as well
Berger et al., 2013 USA [31]	NR	NR
Bonar et al., 2017 USA [32]	13.4% were current smokers, problematic drug use was low and average scores on the AUDIT-C were situated at proposed cut-offs for hazardous drinking.	NR
Brache and Stockwell 2011 Canada [33]	Significant associations between the consumption of AmED and any stimulant drug use (cocaine, crack-cocaine, amphetamines, and crystal meth)	NR
Cecilia et al., 2016 Italy [34]	NR	To stay awake and to treat a hangover
Cobb et al., 2015 USA [35]	NR	To hide the flavor of alcohol, to drink less and get drunk, only mixer available, and stay alert while drinking
de Haan et al., 2012 Netherlands [36]	NR	NR
Eckschmidt et al., 2013 Brazil [37]	NR	NR
Graczyk et al., 2020 USA [38]	NR	Common availability of AmED, taste and effects of drinking AmED (most believing that they can increase alertness, increase energy, reduce sleepiness, and be able to consume more alcohol)
Haas et al., 2017 USA [39]	NR	NR
Johnson et al., 2015 UK [40]	NR	66.5% for the taste, 35.2% to celebrate a special occasion, 45.6% to get drunk
Johnson et al., 2018 UK [41]	NR	NR
Kensinger et al., 2014 USA [23]	NR	To forget your worries, because it helps to enjoy a party, because it helps you when you feel depressed or nervous, to cheer you up when you are in a bad mood, because you like the feeling, because it is exciting, to get high, because it makes social gatherings more fun, to fit in with a group you like, because it improves parties and celebrations, to forget about your problems, because it’s fun
Kurtuncu and Kurt 2021 Turkey [42]	NR	To make the party funnier (2.9%) and to increase the intake of alcohol (2.5%)
Lau-Barraco et al., 2013 USA [24]	The mean global positive change, enhanced sexual performance, physical and social pleasure, relaxation and tension reduction, and arousal and power scores were significantly lower for the Low Alcohol/Low CAB class compared to the High Alcohol/Low CAB class.	NR
Linden-Carmichael and Lau-Barraco 2017 USA [26]	NR	NR
Linden-Carmichael and Lau-Barraco 2017 USA [43]	Impulsivity	NR
Linden-Carmichael and Lau-Barraco 2018 USA [44]	NR	Social, enhancement, and coping motives were unrelated to AmED use
MacKillop et al., 2012 USA [45]	NR	Stay alert longer, more energy to party, get high or “buzzed” quicker
Mallett et al., 2014 USA [46]	Legal, academic and sexual consequences	NR
Mallett et al., 2015 USA [47]	NR	NR
Marczinski et al., 2011 USA [48]	NR	To get drunk and reduce sedation compared to alcohol alone
Marzell et al., 2014 USA [49]	Legal, academic and sexual consequences	NR
Miller et al., 2012 USA [50]	Sexual risk behaviors: casual sex, intoxicated sex and unprotected sex	NR
Norberg et al., 2017 Australia [51]	The feeling lively, happy, and having fun would be associated positively with AmED	NR
O’Brien et al., 2008 USA [52]	Taking advantage of another sexually, riding with an intoxicated driver, being physically hurt or injured, and requiring medical treatment	NR
Oh et al., 2019 South Korea [53]	Missing class, engaging in unplanned sexual activity, having a hangover, doing something you regret, getting behind in school work, arguing with friends, getting hurt or injured, damaging property, being sexually assaulted, getting into trouble with campus or local police, requiring medical treatment	NR
Patrick et al., 2014 USA [54]	Been in a car accident, had a minor injury, had a serious injury, been hospitalized, had surgery or had problems with the police	NR
Patrick et al., 2016 USA [55]	Binge drinking emerged as a strong predictor of AmED use	NR
Price et al., 2010 Canada [56]	Individuals drank significantly more alcohol when it was co-administered with EDs	NR
Sheehan et al., 2016 USA [25]	AmED use quantity was correlated with alcohol use quantity, increase of aggressiveness	NR
Sljivo et al., 2020 Bosnia and Herzegovina [57]	NR	Relaxation
Snipes and Benotsch, 2013 USA [58]	AmEDs consumption was associated with marijuana, ecstasy and cocaine use and with having unprotected sex, sex after having “too much to drink”, sex after drug use and multiple sexual partners in the past 3 months	NR
Snipes et al., 2014 USA [59]	AmED consumption was a significant predictor of patterns of alcohol dependence	NR
Spangler et al., 2018 USA [60]	AmED consumption was associated with ever smokeless tobacco use and cigarette smoking in the past 30 days	NR
Velazquez et al., 2012 USA [61]	AmED consumption was associated with alcohol use	NR
Woolsey et al., 2010 USA [62]	AmED users reported higher average days drinking per week, average number of drinks per occasion, total binge drinking episodes in past year, greatest number of drinks on one occasion, and total number of drinks than alcohol-only consumers	To act aggressively, to be more alert, to feel stronger, to feel sober up quicker and to drive a vehicle were more commonly reported by AmED than by alcohol-only consumers
Woolsey et al., 2015 USA [63]	AmED users are more likely to drive after drinking, drive while knowingly over the blood alcohol content driving limit and to ride with an intoxicated driver	36% reported feeling more confident, 45% felt they could drink more alcohol, 25% felt energy drinks reduce the negative effects of alcohol, 20% felt energy drinks sober them up quicker, and 13% felt that they were more capable to drive
Woolsey et al., 2015 USA [64]	AmED users reported more driving despite knowing they had too much alcohol to drink, driving over the blood alcohol content driving limit, more alcohol average drinks, days drinking (30 day), days drunk (30 day), heavy episodic drinking (30 day), greatest number of drinks on one occasion (30 day), greatest number of drinks on one occasion (12 month), hours of consumption per drinking occasion than alcohol-only users	NR

NR = Not Reported.

## Data Availability

Data is contained within the article.

## Supplementary Materials

The following supporting information can be downloaded at: https://www.mdpi.com/article/10.3390/nu14234985/s1, Figure S1: Funnel Plot Results related to the global prevalence of AmED consumption among undergraduates; Table S1: Detailed search strategy.
