# Supplementary material for: Alcohol Mixed with Energy Drinks (AmED) Use among University Students: A Systematic Review and Meta-Analysis
Source: Nutrients. 2022 Nov 24;14(23):4985. doi: 10.3390/nu14234985 (PMC9737502; doi:10.3390/nu14234985)
Supplement: Supplementary file 1 [file nutrients-14-04985-s001.zip › nutrients-1995553-supplementary.pdf]

Table S1. Detailed search strategy

Database	Search strategy
**Pubmed**	(("energy drink"[All Fields] AND "alcohol"[All Fields]) OR "AmED"[All Fields]) AND ("college students"[All Fields] OR "university students"[All Fields] OR "undergraduate*"[All Fields])
**Scopus**	TITLE-ABS-KEY ("energy drink" AND "alcohol" OR "AmED" AND ("college students" OR "university students" OR "undergraduate*"))
**Web of Science**	TOPIC: ("energy drink" AND "alcohol" OR "AmED" AND ("college students" OR "university students" OR "undergraduate*"))<br>Timespan: All years. Indexes: SCI-EXPANDED, SSCI, A&HCI, CPCI-S, CPCI-SSH,<br>BKCI-S, BKCI-SSH, ESCI, CCR-EXPANDED, IC.

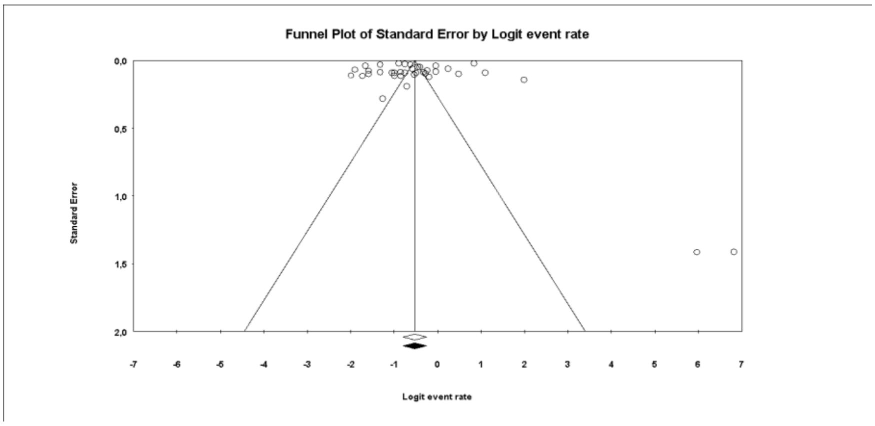

Figure S1. Funnel Plot Results related to the global prevalence of AmED consumption among undergraduates.
